# Electrical stimulation promotes structural plasticity in cortical spheroids by modulating N-cadherin-dependent collective migration and mechanotransduction

**DOI:** 10.1007/s13534-026-00586-9

**Published:** 2026-05-06

**Authors:** Eunmin Ko, Eunyoung Park, Jennifer H. Shin

**Affiliations:** Department of Mechanical Engineering, Korea Advanced Institute of Science and Technology, 291, Daehak-ro, Daejeon, 34141 Republic of Korea

**Keywords:** Electrical stimulation, Cortical spheroids, Neuroplasticity, N-cadherin, Mechanobiology

## Abstract

Electrical stimulation (ES) is a promising tool for brain-computer interfaces and therapeutic neuromodulation, yet its cellular effects on neural tissue remain incompletely understood. Here, we examined the mechanobiological remodeling of 3D cortical spheroids in response to DC electrical stimulation. Prolonged stimulation (60 min) produced a duration-dependent phenotypic shift marked by reduced collective spreading, elevated traction forces, and enhanced neurite outgrowth and complexity, along with increased expression of the plasticity-related immediate-early genes Arc and c-Fos. Higher traction was observed near the spheroid periphery, where IBA1-positive microglia were enriched, suggesting a possible role for microglia in restricting spheroid spreading. This response was accompanied by spatial reorganization of N-cadherin from a peripheral cohesive pattern to a dense, heterogeneous clustered distribution. Pharmacological inhibition of N-cadherin with ADH-1 reversed the migration arrest, supporting a functional role for N-cadherin-mediated adhesion in this phenotype. In parallel, integrin β1 and fibronectin were upregulated and spatially reorganized, indicating substantial remodeling of the adhesion- and ECM-related microenvironment. Together, these findings provide a mechanobiological framework for understanding how electrical stimulation reshapes tissue organization and plasticity-related responses in neural systems, and highlight 3D cortical spheroids as a useful platform for investigating stimulation-induced tissue remodeling.

**Supplementary Information:**

The online version contains supplementary material available at https://doi.org/10.1007/s13534-026-00586-9.

## Introduction

The field of electroceuticals, centered on therapeutic electrical stimulation (ES), has advanced rapidly, particularly in its application to the brain. This interest stems from the ability of electrical stimulation to modulate brain activity by leveraging the intrinsic electrical properties of neural tissue. Unlike pharmacological treatments, which often face limitations in efficacy or specificity, electrical stimulation offers a non-invasive alternative that may improve cognitive and motor function in various neurological disorders [1]. Modalities such as transcranial alternating current (tACS), direct current (tDCS), and magnetic stimulation (TMS) have elicited functional improvements in both healthy and clinical populations; for example, tACS has been reported to enhance memory in older adults, while targeted tDCS has improved performance on salience network-related tasks [2, 3]. In parallel, invasive stimulation paradigms, such as electrocorticography-based brain–machine interfaces with deep brain stimulation, can drive goal-directed behavior in preclinical models [4].

Therapeutic applications have expanded from enhancing normal function to treating pathologies such as Parkinson’s disease and epilepsy, using electroconvulsive therapy (ECT) and deep brain stimulation (DBS) [5–7]. Despite these clinical successes, the underlying mechanisms remain incompletely understood. Although genetics and electrophysiology have characterized brain function, the cellular and biophysical responses of brain cells to imposed electrical inputs remain poorly understood. Specifically, how externally applied fields translate into changes in neurite growth, adhesion, and tissue-scale organization is only beginning to be explored in vitro. Brain cells are highly dynamic; their motility and mechanical interactions, including cell shape, migration, and force generation, are critical for development, homeostasis, and repair [8–13]. These biomechanical characteristics are integrated readouts of cell function that often precede gene or protein expression, offering a complementary lens to molecular analyses. Consequently, investigating the mechanical effects of electrical stimulation may yield more immediate and functionally relevant insights than molecular screens alone.

To address this gap, we examined the cellular mechanical response to electrical stimulation using a three-dimensional (3D) spheroid model. These spheroids, generated from dissociated whole-brain cells cultured using ultra-low-attachment (ULA) plates, exhibit a layered architecture and heterogeneous composition that more faithfully recapitulate native tissue than 2D cultures [14, 15]. By tracking real-time changes in cell migration, force generation, and structural protein expression, we captured both the dynamic and cumulative effects of stimulation. The study further incorporated pharmacological inhibition to probe specific signaling pathways, focusing on N-cadherin’s role in adhesion and cytoskeletal regulation.

In this study, we applied DC electrical stimulation for defined durations and assessed its effects on spheroid behavior. Using quantitative analyses of migration patterns, traction forces, and protein organization, including integrin β1, fibronectin, and N-cadherin, we sought to elucidate how electrical stimulation modulates the physical and biochemical landscapes of brain tissue. Our results suggest that mechanical remodeling is a central mechanism by which brain cells sense and integrate external electrical cues. These insights may advance our understanding of electroceutical interventions and inform the design of more precise neuromodulatory therapies.

## Results

### Electrical stimulation alters cortical spheroid expansion

**Fig. 1:**
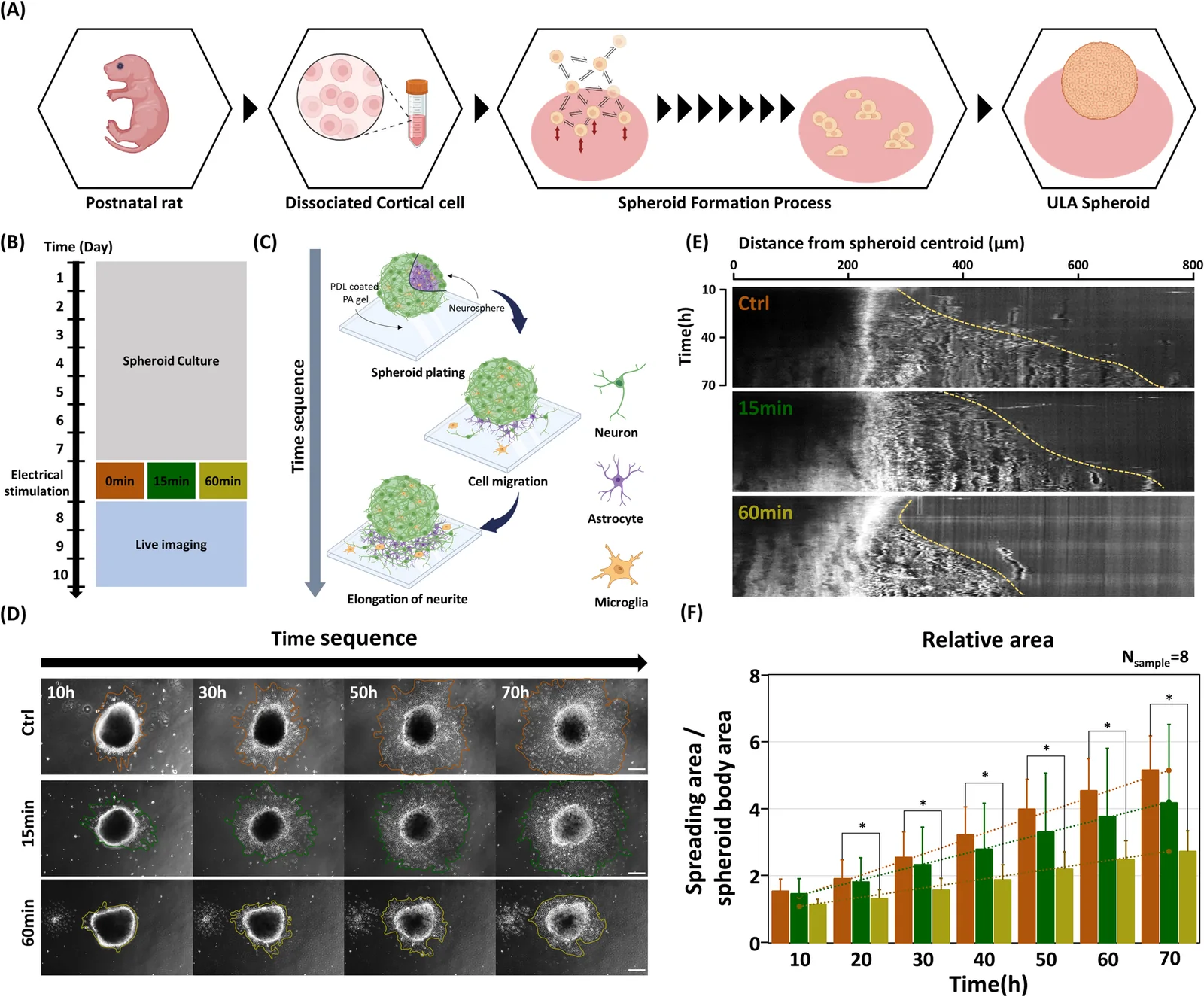
Prolonged electrical stimulation suppresses brain spheroid spreading. **A** Schematic illustration of brain spheroid generation from dissociated postnatal rat cortical cells and subsequent culture in ultra-low attachment (ULA) plates. **B** Experimental timeline showing spheroid culture, electrical stimulation for 0, 15, or 60 min on day 7, and subsequent live imaging for 72 h. **C** Schematic of the spreading assay after spheroid plating, illustrating cell migration and neurite extension from the attached spheroid. **D** Representative live images of spheroid spreading at 10, 30, 50, and 70 h after plating. Dashed outlines indicate the spreading boundary. **E** Representative kymographs showing temporal changes in the radial displacement of the spreading front. **F** Quantification of relative spreading area, expressed as the spreading area normalized to the spheroid body area. Prolonged electrical stimulation (60 min) reduced spheroid spreading compared with the control group, with significant differences observed from 20 h onward. Scale bar, 200 μm. Data are presented as mean ± SD. *P < 0.05 versus control at the indicated time point (one-way ANOVA performed separately at each analyzed time point). *N* = 8 spheroids total

To investigate tissue-level effects of electrical stimulation, we generated 3D spheroids from dissociated postnatal rat cortical cells cultured for seven days under ultra-low-attachment (ULA) conditions (Fig. 1A). These spheroids, comprising neurons, astrocytes, and microglia, were transferred to polyacrylamide (PA) gels and subjected to DC electric stimulation for 0, 15, or 60 min, followed by 72 h of live imaging to track migration dynamics (Fig. 1B and C). Field uniformity in the stimulation region was achieved through the chamber geometry and verified by COMSOL simulations. Agarose salt bridges and Steinberg’s solution were used to electrically couple the electrodes while minimizing electrochemical byproducts (e.g., pH shifts and gas formation) and standardizing ionic conductivity (Supplementary S1A–C).

Qualitative analysis of live-imaging data revealed a duration-dependent migratory response. Although initial spreading (0–10 h) was comparable across all groups, spheroids exposed to 60 min of ES showed a significant reduction in outward expansion beginning at 20 h (Fig. 1D). Kymograph analysis confirmed this inhibitory effect, showing a markedly flattened migration front in the 60-min group, in contrast to the sustained, steep trajectories observed in the control and 15-min cohorts (Fig. 1E).

Quantitative assessment of the relative spreading area (normalized to t = 0) revealed discrete growth kinetics (Fig. 1F). By 70 h, control spheroids showed a 5.1-fold increase in area, whereas the 60-min group expanded only 2.7-fold—representing a 47% reduction relative to controls. The 15-min group showed intermediate behavior (4.2-fold increase). Although its expansion was 35% greater than that of the 60-min group, it did not reach statistical significance compared to the control. These results suggest that short-duration ES is permissive for migration, whereas prolonged stimulation triggers a mechanical state that restricts collective cell migration.

### Electrical stimulation modulates cortical spheroid cell migration

**Fig. 2:**
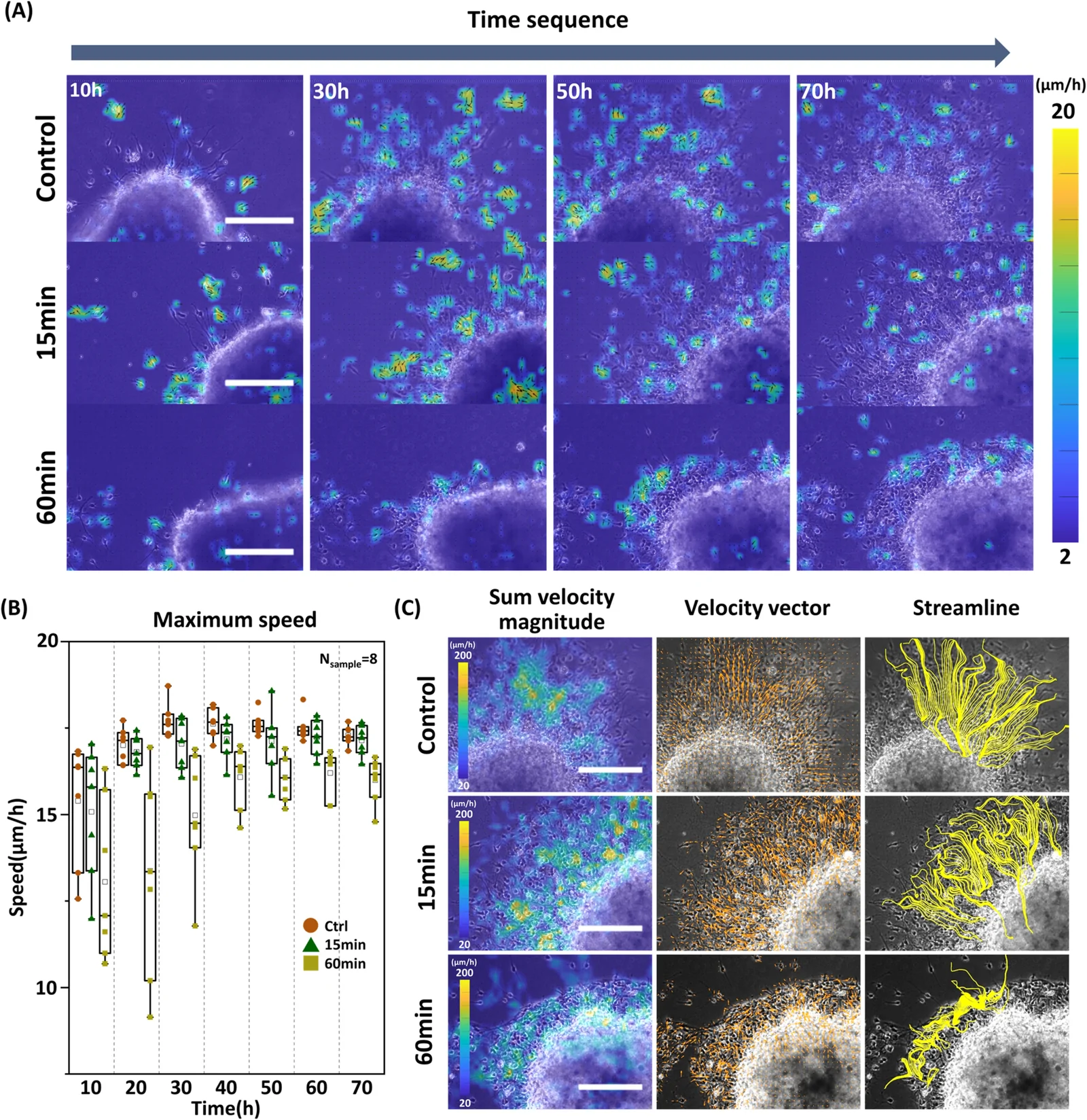
Electrical stimulation alters cell migration dynamics. **A** Time-lapse PIV heatmaps showing migration velocity at indicated time points. **B** Maximum migration speed over time. While all groups showed initial acceleration, the 60-min stimulation group exhibited a prolonged time to reach peak velocity compared to controls. **C** Cumulative migration analysis showing summed velocity magnitude (left), vector fields (middle), and streamline trajectories (right). The 60-min condition displays reduced total movement and confined, curvilinear trajectories, contrasting with the extensive radial spreading observed in the control and 15-min groups. Scale bar: 200 μm. *N* = 8 spheroids

To determine whether the reduction in expansion was driven by altered cellular motility, we quantified migration dynamics using PIV (Fig. 2). Temporal analysis revealed distinct kinetic profiles: in the control and 15-min stimulation groups, migration speed increased rapidly and reached a stable plateau by 30 h. Conversely, the 60-min group showed a marked lag in acceleration, with a transient velocity dip at 20 h and a delayed peak that was not reached until 50 h (Fig. 2A and B).

Quantitatively, the maximum migration speeds in the control and 15-min groups were comparable (17.5 and 17.1 μm/h), whereas the 60-min group had a significantly lower maximum of 16 μm/h. Beyond velocity magnitude, the spatiotemporal organization of movement was fundamentally disrupted. Vector field and streamline analyses revealed coherent, radial trajectories in the control and 15-min groups, indicative of coordinated collective migration. In contrast, the 60-min condition exhibited disordered, curvilinear motion confined to the immediate spheroid perimeter, lacking the directional persistence observed in controls (Fig. 2C).

These findings suggest that prolonged stimulation induces a phenotypic shift that impairs outward polarization. Notably, this restriction in motility coincided with an increase in traction force (Supplementary Fig. S2), particularly at the periphery, where IBA1-positive microglia were enriched. This suggests that although these cells generate substantial mechanical force, it acts as a resistive anchoring force rather than a productive migratory drive, effectively pinning the spheroid to the substrate.

### Electrical stimulation alters ECM composition and structural heterogeneity in cortical spheroids

Given the observed changes in cell motility and traction force, we examined whether electrical stimulation modulated the structural organization of the spheroid’s extracellular matrix (ECM). We evaluated the expression and spatial distribution of integrin β1 and fibronectin, two key proteins governing adhesion and mechanical integrity.

**Fig. 3:**
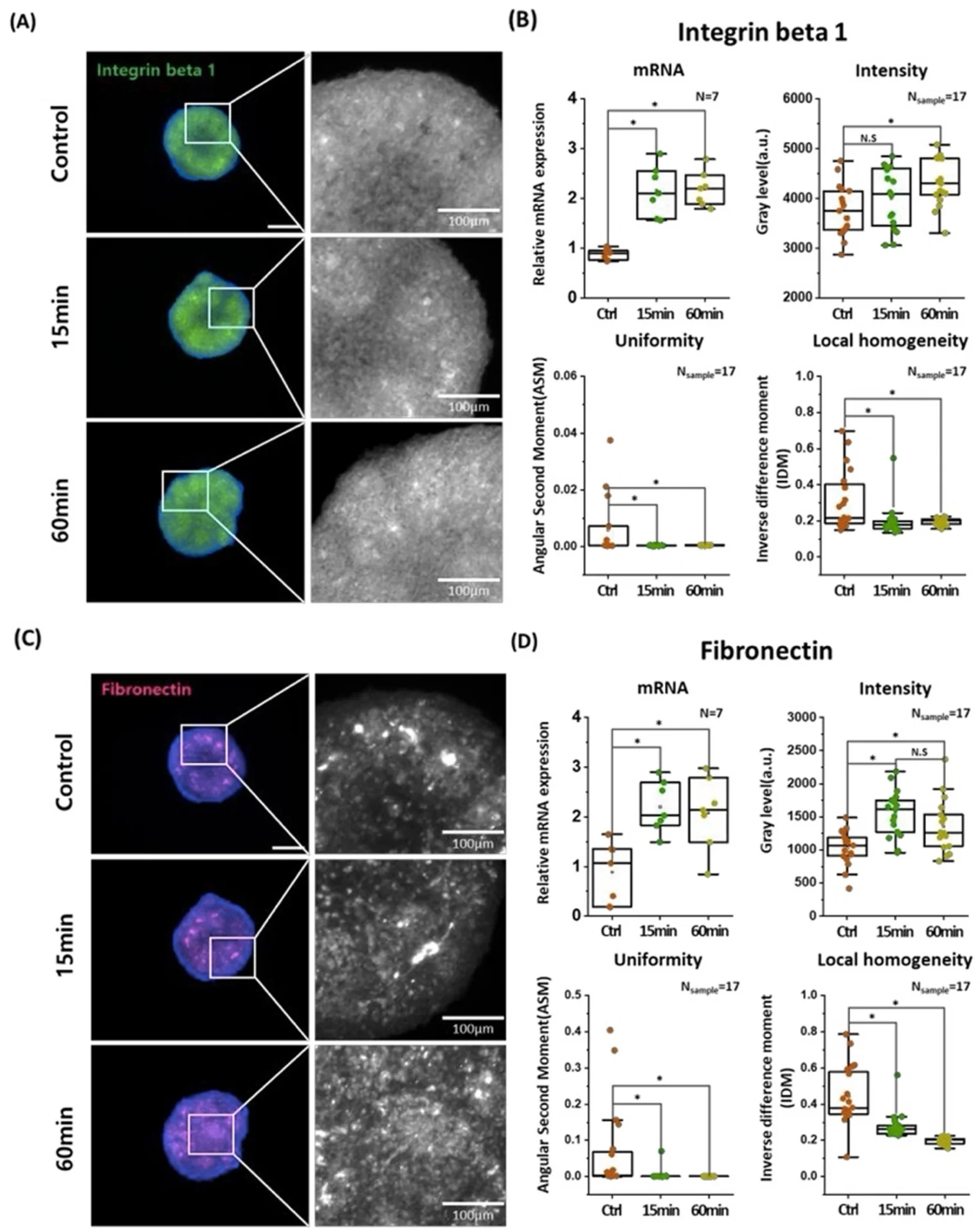
Electrical stimulation alters the expression and spatial organization of integrin β1 and fibronectin in brain spheroids. **A** Representative immunofluorescence images of integrin β1 in spheroid cryosections from the control, 15 min, and 60 min stimulation groups. Boxed regions are shown at higher magnification in the right panels. **B** Quantification of integrin β1, including relative mRNA expression, mean fluorescence intensity, and Haralick texture features representing uniformity (angular second moment, ASM) and local homogeneity (inverse difference moment, IDM). **C** Representative immunofluorescence images of fibronectin in spheroid cryosections from the control, 15 min, and 60 min stimulation groups, with magnified views of the boxed regions shown on the right. **D** Quantification of fibronectin, including relative mRNA expression, mean fluorescence intensity, and texture features (ASM and IDM). Electrical stimulation induced marker- and condition-dependent changes in expression and spatial distribution, with significant alterations in selected comparisons as indicated. Scale bars, 200 μm (main images) and 100 μm (magnified images). Data are presented as mean ± SD. *P < 0.05; *N* = 7 for mRNA expression and *N* = 17 for image-based quantification; one-way ANOVA with pairwise comparisons as indicated

In control spheroids, Integrin β1 exhibited a characteristic annular (“donut-shaped”) distribution, with pronounced peripheral enrichment and a central void (Fig. 3A). Electrical stimulation dismantled this architecture; by 60 min, the central void was obliterated by the emergence of irregular, high-intensity protein subclusters in the spheroid core. This remodeling was associated with significant upregulation of Integrin β1 mRNA (2.45- and 2.49-fold in the 15- and 60-min groups, respectively; Fig. 3B). Protein-level responses followed a duration-dependent pattern: fluorescence intensity increased in both groups but reached statistical significance only in the 60-min group (1.14-fold).

In contrast, fibronectin underwent a distinct structural transition, shifting from an organized, fibrillar network in controls to a fragmented, cluster-like distribution after stimulation (Fig. 3C). Although fibronectin mRNA was significantly upregulated at both time points (~ 2.4-fold), protein expression peaked transiently. Specifically, fibronectin intensity increased significantly at 15 min (1.5-fold) and then settled into a non-significant upward trend at 60 min (1.3-fold; Fig. 3D), suggesting a rapid but perhaps more labile response than that of integrin β1.

To quantitatively assess this topological disorganization, we used Gray-Level Co-occurrence Matrix (GLCM) texture analysis. Electrical stimulation induced a pronounced decrease in the Angular Second Moment (ASM) and Inverse Difference Moment (IDM) for both proteins (Fig. 3C and D, bottom). These reductions confirm a significant loss of spatial uniformity (ASM) and local homogeneity (IDM), indicating a transition from a cohesive, peripherally organized ECM architecture to a heterogeneous, disordered microenvironment. This structural disorder likely serves as a mechanical anchor, facilitating the observed increase in traction forces while simultaneously arresting collective expansion.

### Electrical stimulation enhances neurite extension and neural activation in cortical spheroids

To determine whether the stimulation-induced restriction of cell migration reflected a shift toward neuronal maturation, we assessed neurite outgrowth and neural activation in cortical spheroids. Previous studies have linked electrical stimulation to enhanced neuroplasticity in vivo; consistent with this, we observed distinct morphological remodeling after culturing on poly-D-lysine (PDL)-coated glass.

**Fig. 4:**
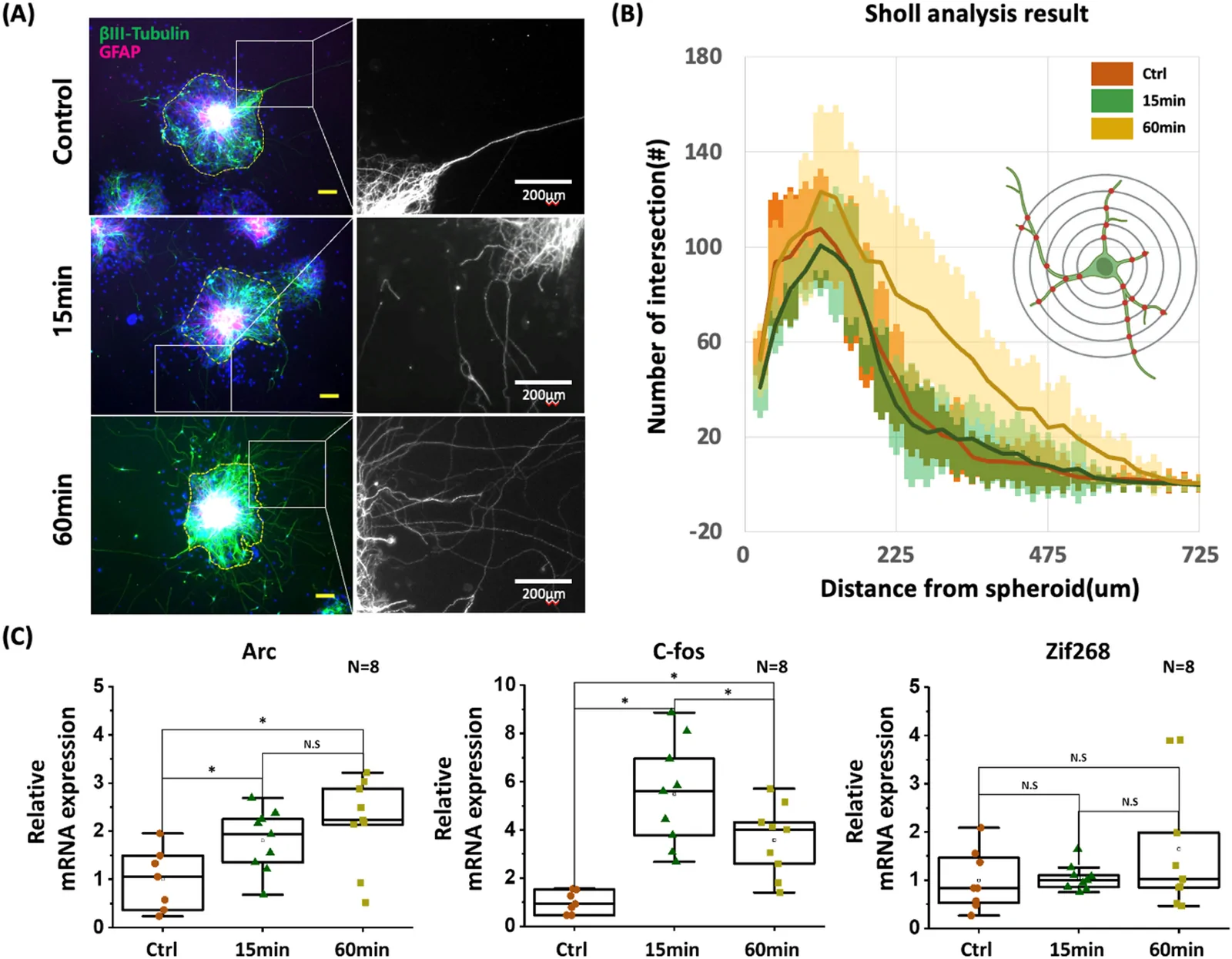
Electrical stimulation modulates neurite outgrowth and immediate early gene expression in brain spheroids. **A** Representative immunofluorescence images of spheroids cultured on PDL-coated glass for 3 days after electrical stimulation. βIII-tubulin is shown in green and GFAP in magenta. Yellow dashed outlines indicate the dense spheroid core, and boxed regions are shown at higher magnification in the right panels. **B** Sholl analysis of neurite outgrowth as a function of distance from the spheroid core. The 60 min stimulation group showed greater neurite complexity and extended neurite distribution compared with the control and 15 min groups. **C** qPCR analysis of immediate early genes (IEGs), including Arc, c-fos, and Zif268. Arc and c-fos expression were significantly altered in selected pairwise comparisons, whereas Zif268 did not show a significant difference among groups, as indicated. Scale bars, 200 μm. Data are presented as mean ± SD in **B** and as box plots with individual data points in **C**. *P < 0.05; *N* = 8; one-way ANOVA with pairwise comparisons as indicated

Immunostaining for βIII-tubulin and GFAP showed that spheroids subjected to 60 min of stimulation developed extensive, high-density neurite networks that extended well beyond the dense cellular core (demarcated by yellow contours in Fig. 4A). Notably, although GFAP-positive astrocytes remained largely sequestered within the consolidated core, neurites exhibited robust radial extension. In contrast, neurite projections in the control and 15-min groups remained largely confined to the immediate vicinity of the spheroid body.

Sholl analysis quantitatively confirmed this increase in morphological complexity (Fig. 4B). Although all groups showed peak neurite density within 100 μm of the spheroid edge, significant divergence emerged at greater radii. Beyond 200 μm, the 60-min group maintained high arborization, with 94 intersections, nearly double the complexity of the control (*N* = 56) and 15-min (*N* = 48) groups. This enhancement persisted up to 625 μm, indicating that prolonged stimulation promotes both axonal elongation and branching.

To assess the functional correlates of this structural remodeling, we profiled expression of immediate-early genes (IEGs) linked to synaptic plasticity (Fig. 4C). Electrical stimulation elicited distinct temporal dynamics across the tested genes. c-Fos mRNA showed rapid, high-magnitude induction, peaking at a 5.5-fold increase in the 15-min group and declining to a 3.6-fold elevation in the 60-min group. Conversely, Arc expression increased steadily and cumulatively, reaching significant elevations in both the 15-min (1.8-fold) and 60-min (2.2-fold) conditions. Zif268 expression trended upward (1.6-fold at 60 min) but did not reach statistical significance. Collectively, these results suggest that 60-min stimulation drives a phenotypic switch, suppressing macroscopic migration and favoring robust neuritogenesis and activation of plasticity-related transcriptional programs.

## N-cadherin inhibition reverses electrical stimulation-induced changes in cortical spheroids

**Fig. 5:**
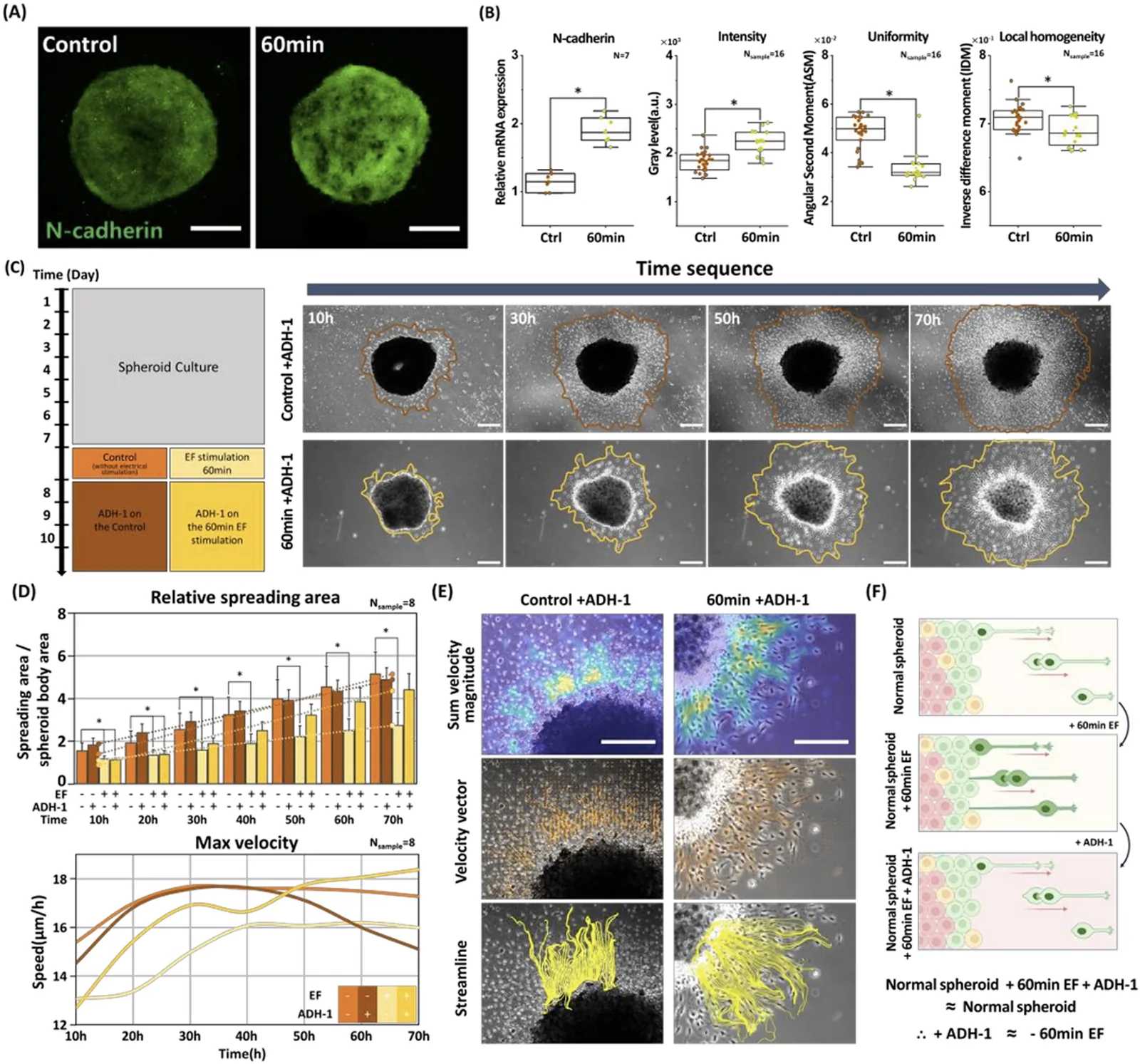
N-cadherin inhibition modulates the spreading phenotype of electrically stimulated brain spheroids. **A** Representative immunofluorescence images of N-cadherin in control and 60 min stimulation groups. **B** Quantification of N-cadherin, including relative mRNA expression, mean fluorescence intensity, and texture features representing uniformity (angular second moment, ASM) and local homogeneity (inverse difference moment, IDM). The 60 min stimulation group showed increased N-cadherin expression and altered spatial texture features compared with the control group. **C** Experimental timeline for ADH-1 treatment and representative live images of spheroid spreading in control and 60 min stimulation groups, with or without ADH-1, shown at 10, 30, 50, and 70 h. Dashed outlines indicate the spreading boundary. **D** Quantification of relative spreading area and maximum velocity over time. ADH-1 altered the spreading dynamics of stimulated spheroids, with significant differences in selected comparisons as indicated. **E** Representative migration maps showing velocity magnitude, vector boundary, and streamline patterns in control + ADH-1 and 60 min + ADH-1 groups. **F** Schematic summary of the proposed effect of ADH-1 on spheroid spreading under normal and electrically stimulated conditions. Scale bars, 200 μm. Data are presented as mean ± SD. *P < 0.05; one-way ANOVA with pairwise comparisons as indicated. *N* = 7 for N-cadherin mRNA, *N* = 16 for N-cadherin image-based quantification, and *N* = 8 spheroids total for spreading analyses

To explore the molecular basis of these changes, we investigated N-cadherin, a critical adhesion molecule that regulates intercellular tension and neural tissue integrity. Immunofluorescence analysis revealed that 60-min stimulation induced profound reorganization of N-cadherin. The typical annular (“ring-like”) distribution observed in controls was replaced by heterogeneous, high-intensity protein subclusters throughout the spheroid core (Fig. 5A). This structural remodeling was confirmed by a 1.66-fold increase in mRNA expression and a 1.20-fold increase in protein fluorescence intensity. Furthermore, GLCM texture analysis quantified this transition; significant decreases in ASM and IDM indicated a loss of spatial uniformity, reflecting a shift from an organized peripheral lattice to irregular central clustering (Fig. 5B).

To determine whether this upregulation was the primary mechanical constraint limiting migration, we treated spheroids with ADH-1, a selective N-cadherin antagonist, following the stimulation protocol (Fig. 5C, left). ADH-1 had negligible effects on non-stimulated controls but effectively rescued the migratory phenotype in the 60-min ES group (Fig. 5C, right). Although the stimulated group initially exhibited a characteristic lag, N-cadherin inhibition allowed the relative spreading area to progressively match control levels by 70 h (Fig. 5D, top). Velocity profiling further confirmed this restoration; the peak migration speed, delayed to 40 + hours in the unmitigated 60-min group, shifted back to the control-like 30-h mark in the presence of ADH-1 (Fig. 5D, bottom). Furthermore, PIV analysis demonstrated that N-cadherin inhibition corrected aberrant motility patterns. The confined, curvilinear trajectories of the stimulated group were reverted to robust, radially oriented streamlines indistinguishable from controls (Fig. 5E).

## Discussions

### Relevance and validity of the 3D spheroid model

Our 3D spheroids were generated from postnatal cortical cells under ultra-low-attachment (ULA) conditions, which promote the spontaneous aggregation of neurons, astrocytes, and microglia into self-organizing microtissues. Unlike reductionist monolayer cultures, this model captures essential features of in vivo brain tissue, including cell–cell signaling, endogenous ECM remodeling, and mechanical reciprocity. The spheroids develop a layered architecture with heterogeneous cell composition that mimics native cortical organization. Although this model lacks vascularization and full laminar complexity, its structural fidelity and functional responsiveness make it an ideal platform for probing the mechanobiological effects of electroceuticals. Importantly, prior work has shown a strong correspondence between in vitro spheroid responses and in vivo outcomes of electrical stimulation [16, 17], supporting the translational relevance of this system. The stimulation setup was designed to minimize non-specific electrochemical artifacts by using agarose salt bridges and Steinberg’s solution, and the 60 min stimulation condition was selected based on prior literature reporting reduced apoptotic responses at this duration.

### Mechanobiological remodeling via N-cadherin: the “brake” on migration

A central finding of this study is that prolonged electrical stimulation (60 min) significantly upregulates and redistributes N-cadherin, converting it from a peripheral cohesion molecule into a barrier to migration. In control spheroids, N-cadherin exhibited an annular (“ring-like”) distribution confined to the spheroid periphery, consistent with the intrinsic self-organization of cortical cells in 3D aggregates, as reported in cortical spheroids and organoids that spontaneously develop radial arrangements and tissue-scale polarity [18, 19]. This pattern parallels radial glia-guided neuronal migration during cortical development, in which spatial polarity and coordinated outgrowth depend on gradients of cell–cell and cell–matrix adhesion [20, 21]. The maintenance of this peripheral adhesion ring in controls supports sustained outward expansion, reaching a 5.1-fold increase in area and a stable velocity plateau of 17.5 μm/h by 30 h. These results indicate that, in the absence of exogenous electrical fields, the spheroids use endogenous adhesion gradients to drive coherent, radially oriented collective migration.

Electrical stimulation disrupted this polarity, causing N-cadherin to redistribute into heterogeneous, high-intensity clusters throughout the spheroid core. Notably, the elevated traction forces observed in the 60-min ES group were not accompanied by increased spheroid expansion, suggesting that the mechanically remodeled state did not support productive outward migration. One possible interpretation is that increased N-cadherin-dependent cohesion contributed to a tissue-level “mechanical brake” that constrained collective spreading. In parallel, traction forces were elevated in peripheral regions enriched for IBA1-positive microglia, raising the possibility that microglia may contribute to this restrictive mechanical state. However, because cell-type-specific force contributions were not directly tested in the present study, including through depletion or inhibition approaches, the role of microglia should be considered suggestive rather than definitive. ADH-1-mediated inhibition of N-cadherin reversed the motility arrest and restored spreading kinetics [20, 22], supporting the conclusion that N-cadherin-dependent adhesion contributes functionally to the migration-restrictive phenotype [23].

### ECM remodeling and selective cellular response

The mechanical remodeling of the spheroid was also accompanied by changes in ECM-related protein organization. Integrin β1 and fibronectin, both implicated in neuronal migration and plasticity-related processes [24–27], were upregulated after electrical stimulation and exhibited marked spatial reorganization. After 60 min of stimulation, both proteins shifted from relatively organized peripheral distributions to more heterogeneous, cluster-like patterns (Fig. 3B), with corresponding decreases in ASM and IDM indicating reduced spatial uniformity and local homogeneity. These results suggest that electrical stimulation reshapes the ECM-related architecture of the spheroid. Although we did not directly assess local matrix stiffness, the emergence of heterogeneous protein clustering may indicate spatial variation in adhesive and mechanical microenvironments. Such changes may contribute to the differential responses observed in the spheroid, where reduced spreading coincided with enhanced neurite outgrowth. However, whether these ECM changes directly restrain glial migration or promote neurite extension will require further mechanistic study.

### Functional plasticity: linking sholl analysis to molecular remodeling

The enhanced neurite complexity observed in Sholl analysis after 60 min of ES is consistent with activity-dependent structural plasticity [28]. First, N-cadherin clustering, although it inhibits migration, may stabilize dendritic arbors and excitatory synapses [25, 29, 30]. Second, remodeling of the fibronectin extracellular matrix and subsequent changes in integrin receptor engagement may have made the microenvironment more permissive for growth cone advance [31]. Third, the sustained elevation of the immediate-early gene Arc indicates that ES engaged transcriptional programs required for long-term synaptic consolidation [32, 33]. Overall, the findings support a model in which ES-induced neurite remodeling is accompanied by coordinated changes in cell adhesion, extracellular matrix interactions, and activity-dependent gene expression.

### Possible signaling pathways

The present findings do not directly delineate the intracellular signaling pathways linking electrical stimulation to the observed structural and transcriptional changes. Nevertheless, prior studies suggest that calcium-sensitive and MAPK-related pathways, including ERK-associated signaling, may contribute to activity-dependent gene expression, adhesion remodeling, and neurite extension in electrically stimulated neural systems [34–38]. Because calcium dynamics and downstream signaling events were not directly assessed here, these pathways are discussed only as possible mechanistic context, rather than as experimentally established mechanisms in this study.

## Conclusion

In summary, this study shows that DC electrical stimulation shifts the balance between collective spheroid spreading and neurite outgrowth in a 3D cortical spheroid model. Prolonged stimulation was associated with reduced collective migration, increased neurite extension, and marked remodeling of adhesion- and ECM-related organization. Our data show that 60 min of stimulation is accompanied by clustering and upregulation of N-cadherin and integrin β1, together with increased structural heterogeneity detected by GLCM texture analysis. These changes coincided with reduced spheroid expansion and altered traction force patterns, suggesting a more mechanically constrained tissue state. In parallel, stimulated spheroids exhibited enhanced neurite outgrowth and increased expression of the immediate-early genes Arc and c-Fos. Pharmacological inhibition with ADH-1 reversed the migration-restrictive phenotype, supporting a functional role for N-cadherin-mediated adhesion in this response. While the underlying signaling pathways and local mechanical properties remain to be directly tested, this work provides a mechanobiological framework for understanding how electrical stimulation reorganizes tissue behavior and plasticity-related responses and supports the use of neural spheroids as an in vitro platform for studying stimulation-induced remodeling.

## Materials and methods

### Animals and cell preparation

Cortical cells were isolated from postnatal day 1–2 Sprague-Dawley rat pups (ORIENT BIO, Korea). The Institutional Animal Care and Use Committee (IACUC) of the Korea Advanced Institute of Science and Technology approved all protocols for mouse experiments. Dissected cortices were enzymatically digested with papain and mechanically dissociated by sequential trituration. After filtration, cells were resuspended in Neurobasal-A medium supplemented with B-27, GlutaMAX, and penicillin-streptomycin. (See Supplementary Methods for details.)

### Cortical spheroid formation

Whole-brain cell spheroids were generated by seeding 3 × 10^5^ dissociated cortical cells per well into 96-well round-bottom ultra-low attachment (ULA) plates (Corning). Cultures were maintained at 37 °C with 5% CO_2_ for 7 days, with the medium replaced every 3–4 days. The use of mixed brain cell types, including neurons, astrocytes, and microglia, allowed for a more physiologically relevant model of brain tissue dynamics. (See the Supplementary Methods for details.)

### Electrical stimulation setup

Electrical current from the power supply was applied indirectly to the cell culture chamber via agar bridges immersed in Steinberg’s solution. The conductive agar bridges were prepared from Steinberg’s solution containing 3% agar powder (Young Sciences, South Korea). A frame for electrical stimulation was fabricated from PDMS.

In this study, several factors informed the selection of electrical stimulation parameters. To replicate the primary experimental target and avoid the complexities of interpreting cellular responses arising from direct interactions between electrical stimulation and cellular components due to membrane penetration, DC stimulation was used exclusively [39, 40]. Simulations of various electrical stimulation modalities indicated that the brain is exposed to electric fields ranging from 1.0 to 3.0 V/cm. Based on this range, 2.0 V/cm was selected as a representative value to mimic brain stimulation conditions [40]. Moreover, according to the paper by M. Hu et al., the apoptotic response of cells was most significantly reduced under 1 h of electrical stimulation [41]. Additional studies confirmed that 1 h of stimulation was sufficient to induce responses from the genomic to the protein level, including gene expression, proliferation, differentiation, and neurite outgrowth [42–44]. Therefore, the experiment was conducted using 1 h of stimulation as the key time point. See Table 1 for the composition of Steinberg’s solution.

**Table 1:** Steinberg’s solution composition

Component	Molarity	Supplier
Tris base	1.4 mM	Usb, USA
NaCl	60 mM	Sigma-Aldrich, USA
KCl	0.7 mM	Sigma-Aldrich, USA
Ca (NO_3_)_2_. 4H_2_O	0.3 mM	Sigma-Aldrich, USA
MgSO_4_.7H_2_O	0.8 mM	Sigma-Aldrich, USA

### Spreading assay

For analysis of cell migratory behavior, PA gels with a stiffness of 1.2 kPa were fabricated. The gels were treated with a diluted sulfo-SANPAH solution in 0.05 M HEPES buffer and exposed to UV light at 365 nm for 30 min. Excess sulfo-SANPAH was removed by washing with 0.05 M HEPES, and the gels were coated with PDL overnight. Cortical spheroids were harvested after seven days and subjected to electrical stimulation. To prevent contact between spheroids during the assay, sufficient spacing was maintained. The PA gels were mounted on an epi-fluorescence microscope equipped with an incubation system, and spreading spheroids were imaged every 30 min for 72 h using phase-contrast imaging to observe cell movements and fluorescent imaging to measure substrate displacements. All live-cell imaging experiments were performed on an Axiovert 200 M (Carl Zeiss) microscope with an incubation system maintained at 37 °C and 5% CO_2_.

### Particle image velocimetry (PIV) analysis for quantifying cell velocity

PIV analysis was performed using the PIV lab software written in MATLAB. It calculates velocity information at multiple grid positions from 2D cross-sectional image pairs. It is based on a discrete Fourier transform technique that computes the correlation matrix from the Fourier transforms of each grid tile and determines the relative position between tiles at different time points. The resulting velocity vectors indicate changes in cell position as a function of both time and position.

### Traction force microscopy

Live cells and embedded fluorescent microbeads in PA gels were imaged with an inverted fluorescence microscope equipped with a 5× objective lens, housed in an enclosed incubation chamber maintained at 37 °C and 5% CO_2_. After the experiments, cells were extracted from the PA gel using 10× trypsin for 1 h, and fluorescent images of the same areas were recorded again as a control. Fluorescent bead images were preprocessed in ImageJ and then analyzed using MATLAB source code provided by J. J. Fredberg’s lab at Harvard T. H. Chan School of Public Health. Traction forces generated by the cells were calculated from bead displacements and the gel’s elastic modulus using unconstrained Fourier transform traction microscopy (FTTM) [45].

### Antibodies

We used purified anti-β-tubulin (Sigma, T8578), GFAP (D1F4Q) XP (Cell Signaling Technology, 12389), purified mouse anti-CD29 (BD Biosciences, 610468), anti-fibronectin (Abcam, ab23750), and N-cadherin monoclonal antibody (Invitrogen, MA1-91128), respectively.

### Immunostaining and imaging

Spheroids were fixed in 4% paraformaldehyde with sucrose, permeabilized, and incubated with primary antibodies against β-III tubulin, GFAP, integrin β1, fibronectin, and N-cadherin. After secondary antibody labeling with Alexa Fluor 488/594, samples were counterstained with DAPI. Imaging was performed on an Axio Observer 7 equipped with an LSM 880 confocal system (Carl Zeiss) (see Supplementary Information for details).

### Cryo-sectioning

Fixed spheroids were cryoprotected in sucrose, embedded in OCT, sectioned at 20 μm with a cryostat, and immunostained using standard protocols (see the Supplementary Information for details).

### RNA isolation and qPCR

Total RNA was extracted with RNAiso Plus (TaKaRa) and cDNA synthesized with iScript (Bio-Rad). qPCR was performed with SYBR Green Supermix on a CFX96 system. Expression levels were normalized to GAPDH and analyzed using the 2^−ΔΔCt method. (See the Supplementary Information for details.)

### Statistical analysis

All experiments were independently repeated three times using separate cell preparations (*n* = 3), which served as biological replicates. A total of 8 spheroids (*N* = 8) were analyzed across these independent experiments. Therefore, the reported spheroid number reflects samples from three biological replicates rather than multiple spheroids from a single preparation. Statistical analyses were conducted using OriginPro 2019 (OriginLab Corporation, Northampton, MA, USA). For live-imaging data acquired during the 72 h post-stimulation period, including spheroid spreading area and cell migration, comparisons among stimulation conditions were performed separately at each analyzed time point using analysis of variance (ANOVA). For endpoint measurements, including gene expression, fluorescence intensity, and image texture-derived parameters, comparisons among groups were performed using one-way ANOVA as appropriate. Pairwise comparisons were conducted according to the experimental design of each dataset and are indicated in the corresponding figures. A significance threshold of *p* < 0.05 was applied to all statistical tests, and results are presented as mean ± standard deviation (SD).

## Supplementary Information

Below is the link to the electronic supplementary material.

Supplementary Material 1

## Data Availability

All data generated or analyzed during this study are included in this published article.
