# Supplementary material for: Electrical stimulation promotes structural plasticity in cortical spheroids by modulating N-cadherin-dependent collective migration and mechanotransduction
Source: Biomed Eng Lett. 2026 May 6;16(5):1273–85. doi: 10.1007/s13534-026-00586-9 (PMC13615234; doi:10.1007/s13534-026-00586-9)
Supplement: Supplementary file 1 [file 13534_2026_586_MOESM1_ESM.docx]

**Supplementary Information**

**Development and analysis of an electrical stimulation system**

**Figure S1**. Illustration and Simulation of the Electrical Stimulation Platform. (A) Schematic depiction of the stimulation generator within the electrical stimulation device, incorporating a function generator, power supply, and amplifier. (B) Schematic representation of the spheroid stimulator in the electrical stimulation device, consisting of an agarose bridge, Steinberg's solution, and a PDMS platform. (C) Simulation results displaying current streamlines and voltage gradients within the platform using COMSOL Multiphysics®. The simulation indicates that the current flow is parallel, and a uniform voltage difference is formed within the area of interest. (Voltage input: 20V, ground: 0V, AC/DC module).

We developed an electrical stimulation platform tailored to the geometry of the spheroids to ensure uniform and reproducible stimulation. The platform consists of two main components: a stimulation generator, which produces the electrical signal, and a spheroid stimulator, which delivers the stimulation directly to the spheroids.

The stimulation generator includes several components: a function generator to define the stimulation period, duration, and waveform; an amplifier to increase the voltage beyond the output limit of the function generator; and a power supply to provide the voltage required for amplifier operation (Fig. S1A). As shown in Fig. S1B, the electrical signal was transmitted from the stimulation generator to the spheroid stimulator through a platinum wire, which was selected for its stable electrochemical properties. Because the direct current used in this study can induce electrolysis, potentially altering medium pH and composition and ultimately causing cell death, we incorporated Steinberg’s solution and an agarose salt bridge. This configuration enabled stable and prolonged electrical stimulation while minimizing electrolysis within the culture medium.

To characterize the electrical environment of the system, computational simulations were performed to analyze current flow and voltage distribution (Fig. S1C). The simulation incorporated the following parameters: medium conductivity of 1.5 S/m, dielectric constant of 80, an input voltage of 20 V, and an output voltage of 0 V. Because no substantial external fluid motion was present in the electrostimulation setup, laminar flow conditions were assumed. The simulation results showed that current streamlines were evenly distributed across the platform. In addition, the voltage gradient decreased linearly from the source to the ground, indicating that the spheroids were exposed to a spatially uniform electric field and a consistent voltage difference across the platform.

**Spheroid's traction force following electrical stimulation**

Because cell migration is driven by physical forces, we hypothesized that the restricted spreading observed in the 60-minute group (Fig. 1) was associated with altered cell–ECM mechanics. Traction force microscopy (TFM) revealed a clear divergence in mechanical behavior (Fig. S2A). Whereas the control and 15-minute groups maintained relatively stable, low-level traction stresses (~5.6–6.1 Pa) throughout the assay, the 60-minute group showed a progressive increase in traction stress, reaching significantly higher forces (8.5 Pa) by 70 hours, corresponding to a ~1.5-fold increase relative to the control group (Fig. S2B).

Because cortical spheroids are heterogeneous aggregates composed of neurons, astrocytes, and microglia, we next sought to identify the cell type contributing most strongly to this high-tension phenotype. Analysis of individual cells at the migration front revealed a distinct subpopulation in the 60-minute group with oscillatory traction dynamics, characterized by periodic bursts of high force generation accompanied by limited net displacement (Fig. S2C). Immunostaining identified these high-traction cells as IBA1-positive microglia (Fig. S2D), whereas βIII-tubulin-positive neurons and GFAP-positive astrocytes contributed comparatively little to the peak traction signals.

These findings suggest that prolonged electrical stimulation induces a mechanically distinct microglial state associated with increased substrate engagement. Rather than serving as leading cells that promote outward migration, these microglia may act as local mechanical anchors at the spheroid periphery. Their elevated traction forces may generate resistive drag that constrains the outward translocation of neighboring neuronal and glial populations.

**Figure S2.** 60-minute electrical stimulation induces an increase in traction force. (A) Phase image with traction force maps at 10- and 70-hours post-control, 15-minute, and 60-minute stimulation. (B) Quantitative traction magnitude results for the control, 15-minute, and 60-minute stimulation groups at 70 hours after re-culturing. Across all conditions, elevated traction values were observed in the spheroid body area, with the 60-minute stimulation condition showing a significantly higher traction value than the others. (C) A detailed traction map highlights cells with discernible traction values. (D) Immunostaining for IBA1 confirms that the cells inducing significant traction changes in the 60-minute sample were microglia (green: IBA1, scale bar: 200μm, scatter plot with boxplot, N_sample_ denotes the number of regions of interest, p*<0.05, analyzed by one-way ANOVA compared with control).

**SUPPLEMENTARY METHODS**

**Isolation of prenatal rat cortical cells.**

Primary cortical tissues were harvested from postnatal day 1–2 Sprague-Dawley rat pups (DBL, South Korea). Tissue dissociation was performed following established protocols [46, 47]. Briefly, dissected cortical tissues were minced into small fragments and incubated in papain solution (Worthington, USA) at 37℃ for 10 minutes. After enzymatic digestion, the tissues were sequentially triturated with 1000-, 100-, and 10-µl pipette tips, each for 20 repetitions, in Dulbecco’s Modified Eagle’s Medium (DMEM; WELGENE, South Korea) supplemented with 10% fetal bovine serum (FBS; Gibco, UK) and 1% penicillin-streptomycin (Gibco, UK). The resulting suspension was filtered through a 40-µm cell strainer to remove debris. Cells were collected by centrifugation at 1500 rpm for 3 minutes, and the supernatant was discarded. The resulting cortical cell pellet was resuspended and cultured in Neurobasal-A medium (Gibco, UK) supplemented with 2% B-27 supplement (Gibco, UK), 1% GlutaMAXTM (Gibco, UK), and 1% penicillin-streptomycin (Gibco, UK).

**Cortical spheroid formation**

The characteristics of spheroids are influenced by the properties of the culture substrate, a phenomenon that also applies to brain cells [15, 17]. To better replicate the complexity of the in vivo brain environment, we employed spheroids derived from whole postnatal rat brain cells rather than purified or lineage-specific cell populations. While single-cell-type models offer analytical simplicity, they cannot recapitulate the dynamic cell–cell interactions, paracrine signaling, and contact-mediated feedback mechanisms intrinsic to brain tissue function. In contrast, our mixed-cell spheroid system incorporates neurons, astrocytes, microglia, and other glial cells, enabling more physiologically relevant intercellular communication. This design allows us to capture emergent multicellular behaviors in response to electrical stimulation that would not be observable in monoculture systems, particularly collective migration dynamics, traction force distribution, and synapse-related remodeling. Furthermore, spheroid characteristics are well known to depend on the culture environment, particularly the substrate. Given that ultra-low attachment (ULA) surfaces have been shown to support the formation and functional maintenance of whole brain spheroids[14], we adopted the ULA-based culture method as described in previous studies. The spheroid culture procedure is briefly described below. Dissociated cortical cells were seeded onto a 96-well round-bottom ultra-low attachment (ULA) plate (Corning, US) at a density of 3 × 10^5^ cells per well to generate spheroids. Cultures were maintained at 37 °C and 5% CO_2_ for 7 days, with half the culture medium replaced with fresh medium every three to four days.

**Cryo-sectioning of cortical spheroids and immunostaining.**

Cortical spheroids were fixed with 4% v/v paraformaldehyde and 8% w/v sucrose in phosphate-buffered saline (PBS) overnight and washed in PBS for 1 hr, three times. The spheroids were then sequentially immersed in 15% and 30% sucrose in PBS for 3 hours per step at room temperature. Samples were embedded in the Tissue-Tek® O.C.T.TM compound (SAKURA, US) and stored at -80℃. Frozen samples were sectioned into approximately 20μm-thick slices using a Leica CM3050 S cryostat (Leica, Germany) and placed on Histobond adhesive glass slides (Marienfeld, Germany). Spheroid sections on adhesive glass slides were dried at room temperature for 30 min and washed twice with PBS. Dried sections were permeabilized with 0.1% Triton X-100 in PBS for 15 min, followed by two washes in PBS. Samples were then blocked in 3% BSA solution with 0.1% Triton X-100 for 1 hr and washed twice with PBS. Incubation with primary antibodies was performed overnight at 4℃. Following two washes in PBS for 30 min, samples were stained with secondary antibodies (Alexa Fluor 488 or 594) for 1 h at room temperature. Sections were washed twice with PBS for 5 min and incubated in DAPI solution for 5 min. Immunohistochemical tissues were imaged with an Axio Observer 7 (Carl Zeiss, Germany) equipped with an Axiocam 702 mono camera, and Nissl-stained tissues were imaged with an Axio Observer 7 equipped with an Axiocam 305 color camera. Confocal z-stack images were acquired with LSM 880 (Carl Zeiss, Germany).

**RNA isolation and quantitative polymerase chain reaction (Quantitative PCR) analysis.**

Cortical spheroids were lysed with RNAiso Plus reagent (TaKaRa, Japan), and total RNA was extracted according to the manufacturer’s instructions. Briefly, lysed samples were incubated with chloroform, then centrifuged (12,000g, 15 min, 4℃). The supernatant was incubated with isopropanol and centrifuged (12,000g, 15 min, 4℃). After removing the supernatant, the pellet was washed with 75% cold ethanol and centrifuged (7,500g, 5 min, 4℃). Extracted RNA pellets were resuspended in ultrapure water (WELGENE, South Korea), and concentrations were measured using a NanoDrop 1000 Spectrophotometer (ThermoScientific, USA). Reverse transcription of RNA to complementary DNA (cDNA) was performed using the iScript cDNA synthesis kit (Bio-Rad, US). Quantitative PCR analysis was performed using primers and SYBR Green Supermix (Bio-Rad, US) on a CFX96TM Real-Time PCR Detection System (Bio-Rad, US). Fold expression was calculated using the 2−ΔΔCτ formula. Gene expression levels were normalized to the housekeeping gene glyceraldehyde-3-phosphate-dehydrogenase (GAPDH). The primer sequences used in this study are shown in Table S1.

**Table S1.** Primer for quantitative PCR

| Gene | Forward sequence  (5’ to 3’) | Reverse sequence  (5’ to 3’) |
| --- | --- | --- |
| Gapdh | GGC AAG TTC AAT GGC ACA GT | TGG TGA AGA CGC CAG TAG ACT C |
| Arc | GCC GCC AAA CCC AAT GTG A | GTC GCC CGT GGG CAC ATA G |
| C-fos | AGC ATG GGC TCC CCT GTC A | GAC ACC AGA GTG GGC TGC A |
| Zif268 | AAT CCC CCT TCG TGA CTA CC | GCA AGG ATG GAG GGT TGG |
| Integrin beta 1 | GGA GAA AAC TGT GAT GCC ATA CAT | TGG GCT GGT ACA GTT TTG TTC A |
| Fibronectin | GAA TCA GCA GTG CCA AAG TGG A | CAC CGT TGT TGA CGT GAA ATG AG |
| N-cadherin | GCA CCA GGT TTG GAA TGG G | CAT GTT GGG AGA AGG GGT G |
